# Cloning, Expression, Purification, and Characterization of Lactate Dehydrogenase from *Plasmodium knowlesi*: A Zoonotic Malaria Parasite

**DOI:** 10.3390/ijms25115615

**Published:** 2024-05-22

**Authors:** Jae-Won Choi, Min-Ji Choi, Yeon-Jun Kim, So Yeon Kim

**Affiliations:** 1 Department of Biomedical Science, Cheongju University, Cheongju 28160, Republic of Korea; 2 Department of Biopharmaceutical Sciences, Cheongju University, Cheongju 28160, Republic of Korea; 3 Department of Dental Hygiene, Cheongju University, Cheongju 28503, Republic of Korea

**Keywords:** *Plasmodium knowlesi*, lactate dehydrogenase, Rosetta(DE3), overexpression

## Abstract

*Plasmodium knowlesi* is the only *Plasmodium* that causes zoonotic disease among the *Plasmodium* that cause infection in humans. It is fatal due to its short asexual growth cycle within 24 h. Lactate dehydrogenase (LDH), an enzyme that catalyzes the final step of glycolysis, is a biomarker for diagnosing infection by *Plasmodium* spp. parasite. Therefore, this study aimed to efficiently produce the soluble form of *P. knowlesi* LDH (PkLDH) using a bacterial expression system for studying malaria caused by *P. knowlesi*. Recombinant pET-21a(+)-*PkLDH* plasmid was constructed by inserting the *PkLDH* gene into a pET-21a(+) expression vector. Subsequently, the recombinant plasmid was inserted into the protein-expressing *Escherichia coli* Rosetta(DE3) strain, and the optimal conditions for overexpression of the PkLDH protein were established using this strain. We obtained a yield of 52.0 mg/L PkLDH from the Rosetta(DE3) strain and confirmed an activity of 483.9 U/mg through experiments. This methodology for high-efficiency PkLDH production can be utilized for the development of diagnostic methods and drug candidates for distinguishing malaria caused by *P. knowlesi*.

## 1. Introduction

Malaria is endemic in tropical and subtropical regions and is the most common febrile infectious disease worldwide. Malaria is caused by the *Plasmodium* spp. parasite, which is transmitted through the bite of a female *Anopheles* mosquito [1]. The representative malarial parasites that cause infections in humans are *P. falciparum, P. vivax, P. malariae, P. ovale, and P. knowlesi* [1,2]. Of these, only *P. knowlesi*, which infects macaque monkeys, causes zoonotic diseases [2,3]. The first report of a *P. knowlesi*-induced infection in humans was reported in the Kapit Division of Sarawak, Malaysia, in 2004 [3,4]. Recently, infection rates in Asian countries, such as Thailand, Singapore, Indonesia, Vietnam, and the Philippines, have increased [5]. To reduce the risk of malaria caused by *P. knowlesi*, the World Health Organization (WHO) has been monitoring the status *P. knowlesi* malaria infections around the world since 2010. Malaria caused by *P. knowlesi* is known to be transmitted to humans by macaque monkeys and *Anopheles* mosquitoes [6,7,8], and the zoonotic characteristic of this infection hinders efforts to eradicate malaria [9]. Therefore, efforts should be made to control this parasite.

*P. knowlesi* can be fatal, owing to its short asexual growth cycle (within 24 h), which is the shortest of all *Plasmodium* species in the erythrocytic stage [5,10]. The WHO recommends a parasite-specific diagnosis of individuals suspected of malaria before treatment to prevent drug abuse and combat antimalarial drug resistance. Therefore, the WHO emphasizes the need to invest efforts in developing tools that can detect and distinguish the five *Plasmodium* species that cause infections in humans [1]. Because *P. knowlesi* has been studied for a relatively short period of time compared to other malaria parasites, there is a lack of technology to specifically diagnose or treat *P. knowlesi* from other malaria parasites [11,12,13]. Therefore, studies on the diagnosis and treatment of infections caused by *P. knowlesi* should be prioritized.

Blood smear tests using a microscope is the most widely used method for malaria diagnosis [14,15]. However, the trophozoites of *P. knowlesi* are similar to those of *P. malariae* and *P. falciparum* [12,14,15]. Therefore, it is difficult to accurately distinguish *P. knowlesi* from *P. falciparum* and *P. malariae* by microscopy in countries where zoonotic diseases are prevalent. The identification of *Plasmodium* species is important because the methods of diagnosis and treatment vary depending on the infected *Plasmodium* species [16]. Gene amplification by polymerase chain reaction (PCR) using primers specific for *Plasmodium* is not widely used in malaria-endemic regions because of a lack of facilities and time-consuming procedures [11]. Rapid antigen testing (RAT) is widely used in malaria-endemic regions because of its ability to quickly identify infectious agents in the field without medical equipment or pathologists [17,18]. A commercialized RAT has been reported to detect *Plasmodium* spp. infections in humans [19]. However, research on RAT that can specifically diagnose *P. knowlesi* by distinguishing it from other *Plasmodium* species is limited. Therefore, further research should be conducted on RAT for the differential diagnosis of *P. knowlesi* malaria.

The antigens mainly used in RAT for the diagnosis of malaria include lactate dehydrogenase (LDH) [20,21], histidine-rich protein-2 (HRP-2) [22], glutamate dehydrogenase [23], and aldolase [24]. LDH exclusively catalyzes the final step of glycolysis and converts pyruvate and NADH to lactate and NAD^+^, respectively [25]. The *Plasmodium* parasite relies only on glycolysis for energy production as the tricarboxylic acid cycle is lacking in the erythrocytic stage; thus, LDH is particularly abundant in erythrocytic trophozoites [20,26]. Therefore, LDH in the blood of infected patients is a suitable biomarker for diagnosing malaria [20,21]. This study aimed to produce a soluble form of *P. knowlesi* LDH (PkLDH) using a bacterial system to diagnose and develop treatment strategies for malaria. Herein, we report on methods for producing soluble PkLDH at high yields using a bacterial expression system and describe the biochemical characteristics of the produced PkLDH.

## 2. Results

### 2.1. Homology Analysis of Amino Acid Sequence of PkLDH with Other Plasmodium spp. LDH

**Figure 1:**
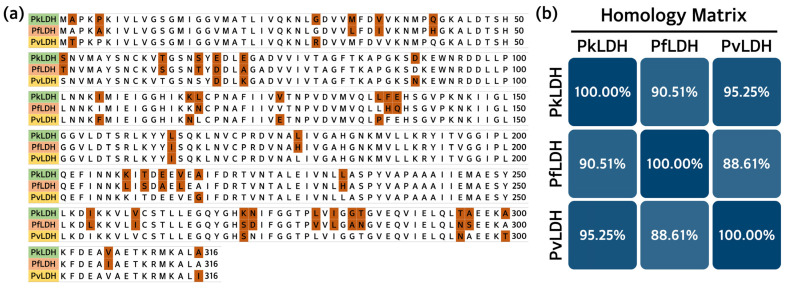
Amino acid identity of major *Plasmodium* spp. LDH. (**a**) Table comparing amino acid sequence (316 amino acids) of PfLDH and PvLDH with those of PkLDH, respectively. Red boxes indicate areas where PkLDH differs from the amino acids of PfLDH and PvLDH. (**b**) Percent identity of amino acid sequence of PkLDH with PfLDH and PvLDH. PfLDH and PvLDH exhibited 90.51% and 95.25% identity with PkLDH, respectively. PfLDH, *Plasmodium falciparum* lactate dehydrogenase; PvLDH, *Plasmodium vivax* lactate dehydrogenase; PkLDH, *Plasmodium knowlesi* lactate dehydrogenase.

The *PkLDH* cDNA (GeneBank No. JF958130.1) sequence obtained from the National Center for Biotechnology Information (NCBI) was compared and analyzed with the sequences of the *PfLDH* gene (M93720.1) and *PvLDH* gene (JN547223.1). Basic Local Alignment Search Tool (BLAST) analysis revealed 78.78% identity with *PfLDH* and 81.87% identity with the *PvLDH* gene. The amino acid sequences of PkLDH were compared and analyzed using PfLDH and PvLDH. Analysis was performed using the UniProt alignment database, and the results showed 90.51% identity with PfLDH and 95.25% identity with PvLDH (Figure 1).

### 2.2. Construction of Recombinant Plasmid for PkLDH Expression and Purification

**Figure 2:**
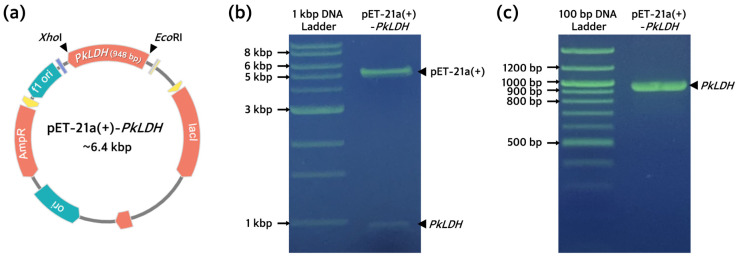
Construction of pET-21a(+)-*PkLDH* for recombinant PkLDH production. (**a**) Plasmid map of pET-21a(+)-*PkLDH*. *PkLDH* gene from the pUC-IDT-*PkLDH* was inserted into the *Xho*I and *Eco*RI restriction sites of pET-21a(+). Recombinant plasmid was introduced into DH5α strain for subcloning. (**b**) An agarose gel image of digested plasmid construct from DH5α strain with *Xho*I and *Eco*RI restriction enzymes. Gel electrophoresis was performed on a 0.8% agarose gel. (**c**) An agarose gel image of PCR product of plasmid construct using DH5α strain with *PkLDH*-specific primers. Electrophoresis was performed on a 1.2% agarose gel.

In this study, *PkLDH* genes were obtained by PCR from the pUC-IDT-*PkLDH* plasmid. The *PkLDH* gene was introduced into the expression vector pET-21a(+) to construct recombinant pET-21a(+)-*PkLDH* plasmid (Figure 2a). After transformation using *Escherichia coli* DH5α strain, plasmid digestion was performed and purified using *Xho*I and *Eco*RI restriction enzymes to confirm that the *PkLDH* gene was inserted into the pET-21a(+) vector. Digested plasmid showed bands of ~5.4 kbp and ~1.0 kbp for the pET-21a(+) and *PkLDH* genes, respectively (Figure 2b). Additionally, 1 ng of pET-21a(+)-*PkLDH* was subjected to PCR, which confirmed the presence of a single band of ~1.0 kbp (Figure 2c). Finally, the *PkLDH* sequence of the recombinant plasmid was verified by capillary automated electrophoresis-based DNA sequencing analysis using T7 promoter- and T7 terminator-specific primers. BLAST analysis confirmed that it was 100% identical to the *PkLDH* gene (JF958130.1). Therefore, we confirmed that the pET-21a(+)-*PkLDH* plasmid was successfully constructed to produce recombinant PkLDH.

### 2.3. Optimization of Recombinant PkLDH Overexpression

**Figure 3:**
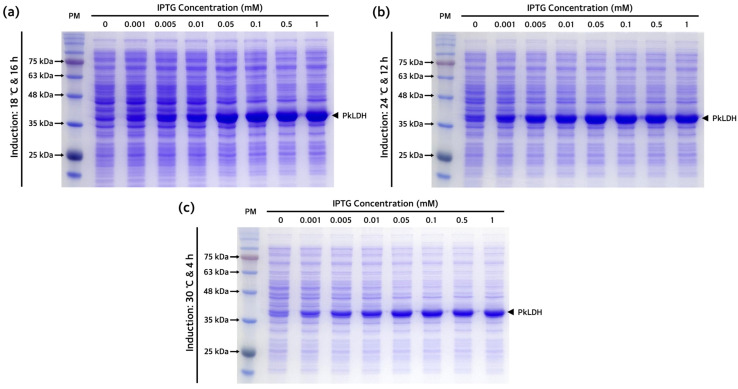
Overexpression pattern of PkLDH according to temperature and IPTG concentration. (**a**) A polyacrylamide gel image after sodium dodecyl sulfate-polyacrylamide gel electrophoresis (SDS-PAGE) of lysate from the transformed *E. coli* Rosetta(DE3) induced at 18 °C for 16 h. (**b**) A polyacrylamide gel image of lysate from the transformed *E. coli* Rosetta(DE3) induced at 24 °C for 12 h. (**c**) A polyacrylamide gel image of lysate from transformed *E. coli* Rosetta(DE3) induced at 30 °C for 4 h. All three experiments were performed under same SDS-PAGE conditions. Overexpression of PkLDH was induced using different concentrations of IPTG (0, 0.001, 0.005, 0.01, 0.05, 0.1, 0.5, and 1 mM). SDS-PAGE was performed on a 12% separating gel. Arrow to the right of each gel indicates overexpressed PkLDH protein.

**Figure 4:**
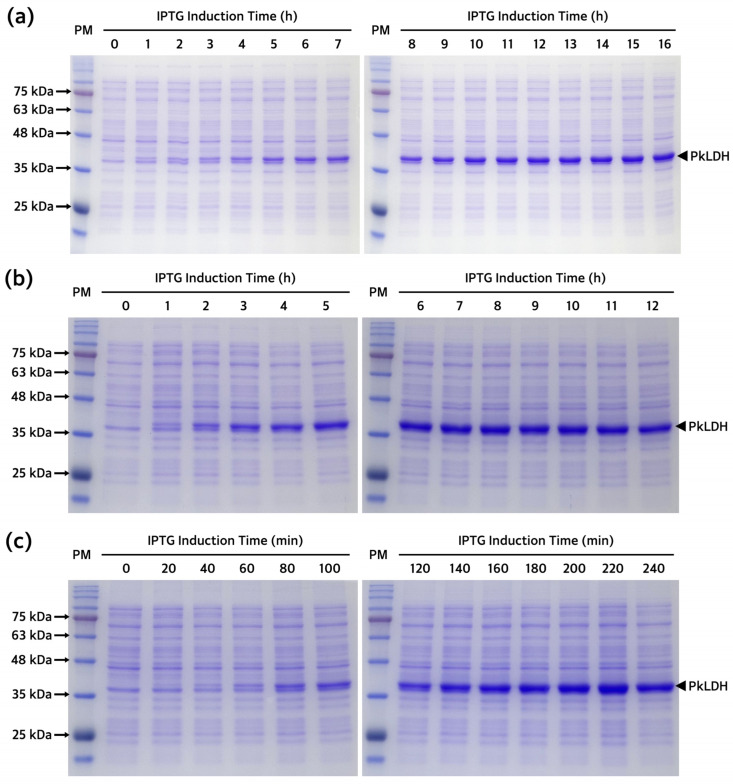
Overexpression pattern of PkLDH according to time at different temperatures. (**a**) A polyacrylamide gel image after SDS-PAGE of lysate from the transformed *E. coli* Rosetta(DE3) induced at 18 °C for 16 h. Bacterial cells collected hourly. (**b**) A polyacrylamide gel image of lysate from the transformed *E. coli* Rosetta(DE3) induced at 24 °C for 12 h. Bacterial cells were collected every hour. (**c**) A polyacrylamide gel image of lysate from transformed *E. coli* Rosetta(DE3) induced at 30 °C for 4 h. Bacterial cells were collected every 20 min. All three experiments were performed under the same SDS-PAGE conditions. Overexpression of PkLDH was induced using 0.1 mM of IPTG. SDS-PAGE was performed on a 12% separating gel. Arrow to right of each gel indicates the overexpressed PkLDH protein.

The subcloned pET-21a(+)-*PkLDH* plasmid construct present in *E. coli* DH5α was introduced into the *E. coli* Rosetta(DE3) strain, which is specialized for expressing eukaryotic proteins. Several overexpression conditions were explored for the optimal production of PkLDH. We confirmed the overexpression pattern of PkLDH at different temperatures and isopropyl β-D-1-thiogalactopyranoside (IPTG) concentrations (Figure 3). Analysis of the conditions for PkLDH overexpression at 18 °C, 24 °C, and 30 °C confirmed that overexpression was successful under all three conditions. Additionally, it was confirmed that sufficient overexpression was achieved when all cases were treated with IPTG at a concentration of 0.1 mM. Moreover, analysis of the overexpression pattern by time according to temperature by treating with 0.1 mM IPTG also confirmed that at least 12 h of induction was required at 18 °C (Figure 4a). Further, it was confirmed that at least 6 h and 3 h of induction were required at 24 °C and 30 °C (Figure 4b,c), respectively. 

### 2.4. Purification and Biochemical Analysis of Recombinant PkLDH

**Figure 5:**
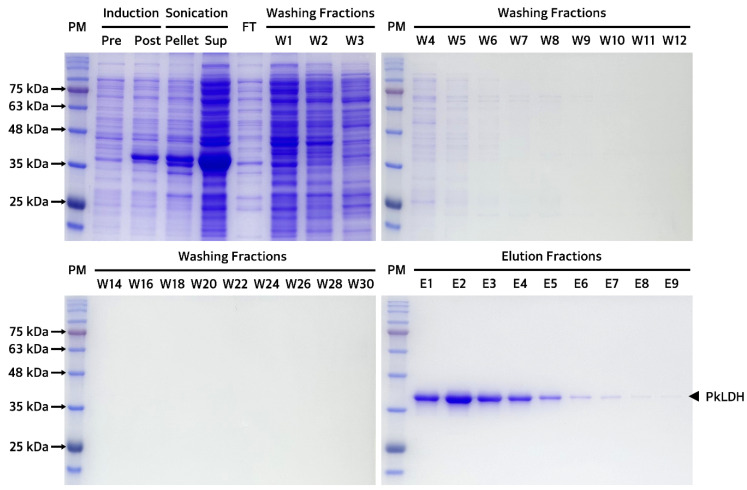
Purification process of recombinant PkLDH by affinity chromatography using a Ni-NTA resin. SDS-PAGE results for all fractions of affinity chromatography starting from sample preparation process for purification of recombinant PkLDH. Gel images show differences in PkLDH overexpression pre-induction (Pre) and post-induction (Post) sample. Additionally, pellet and supernatant (Sup) obtained by centrifugation after bacterial lysis show that most of the PkLDH is present in the supernatant. FT represents the flow-through fraction obtained by loading a bacteria lysate sample. W1 to W30 represent washing fractions that were sequentially passed through Ni-NTA resin by adding washing buffer. E1 to E9 represent elution fractions that were sequentially passed through Ni-NTA resin by adding elution buffer. In all gels, SDS-PAGE was performed on a 12% separating gel under denaturation conditions.

Recombinant PkLDH was overexpressed using 0.1 mM of IPTG for 16 h at 18 °C. Bacterial cell pellets were collected and completely dissolved in an equilibrium buffer containing lysozyme and protease inhibitors. After centrifugation, it was confirmed that the majority of PkLDH was present in the supernatant rather than in the pellet (Figure 5), indicating that PkLDH shows soluble expression rather than an insoluble expression called inclusion bodies. After the nickel-nitrilotriacetic acid (Ni-NTA) resin reached equilibrium with the equilibrium buffer, the bacterial lysate was loaded onto a column packed with Ni-NTA resin. A washing buffer equivalent to 30 times the volume of Ni-NTA resin was used to remove impurities and proteins showing non-specific binding. Finally, recombinant PkLDH, which was strongly bound to Ni^+^, was eluted by adding an elution buffer equivalent to 10 times the volume of resin. The eluted fractions were collected and dialyzed three times using phosphate buffered saline (PBS) to obtain recombinant PkLDH.

**Figure 6:**
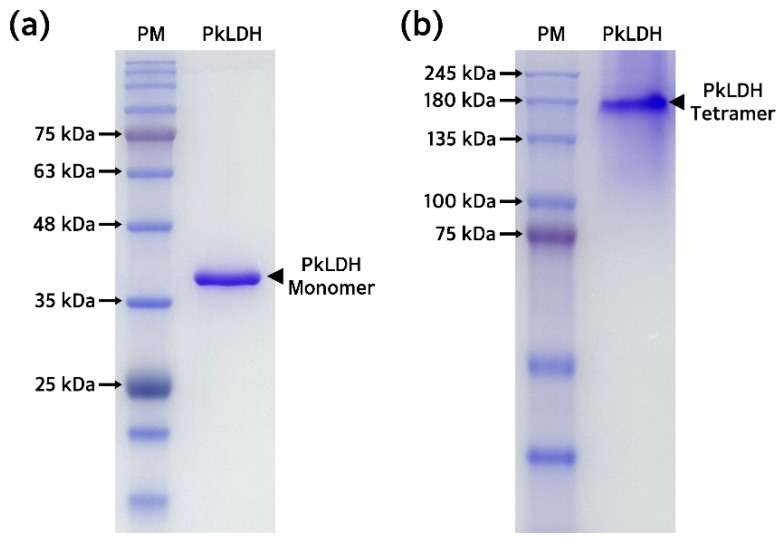
Polyacrylamide gel electrophoresis for confirmation of PkLDH purity and tetramer formation. (**a**) A polyacrylamide gel image confirming the monomer form and purity of PkLDH. SDS-PAGE was performed on a 12% separating gel under denaturation (reducing) conditions. (**b**) A polyacrylamide gel image confirming tetramer formation and molecular weight of the PkLDH tetramer. At this time, PkLDH was prepared under native (non-reducing) conditions in a buffer without SDS. PAGE was performed on a 10% separating gel without SDS.

Purified recombinant PkLDH was quantified using the bicinchoninic acid (BCA) protein assay. Afterwards, to confirm the purity and the molecular weight of the monomer, sodium dodecyl sulfate-polyacrylamide gel electrophoresis (SDS-PAGE) was performed under denaturation (reducing) conditions using a 12% polyacrylamide gel by loading 2 μg of PkLDH. This revealed a single band around ~35.0 kDa, indicating >99.0% purity and successful purification of PkLDH (Figure 6a). Additionally, PAGE was performed using the same amount of PkLDH under native (non-reducing) conditions. A single band was identified between 135 and 180 kDa in the gel, suggesting that PkLDH was well formed as a tetramer, which is its original characteristic under native conditions (Figure 6b).

### 2.5. Measurement of PkLDH Activity

**Figure 7:**
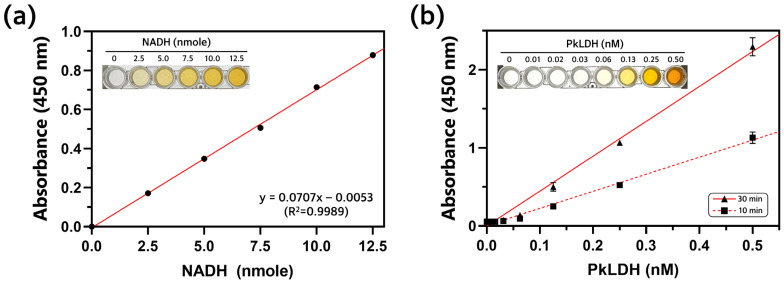
Colorimetric assay for measurement of PkLDH activity. (**a**) Standard curve by mixing different amounts of NADH (0, 2.5, 5.0, 7.5, 10.0, and 12.5 nmole) and substrate mixture. The total reaction volume was 100 μL and absorbance was measured at 450 nm and 37 °C. Inset image shows the yellow product according to different amounts of NADH, and the greater the amount of NADH, the more products there are, so the yellow color becomes darker. (**b**) Colorimetric LDH assay according to different final concentrations of PkLDH (0, 0.01, 0.02, 0.03, 0.06, 0.13, 0.25, and 0.50 nM). Total reaction volume was 100 μL, and absorbance was measured at 450 nm and 37 °C. Absorbance was measured at 10 min and 30 min after reaction for each concentration in triplicates.

A colorimetric LDH assay was performed to determine the activity of recombinant PkLDH in the presence of a substrate. This assay assumes that LDH converts NAD^+^ to NADH when lactate is degraded into pyruvate, and the NADH generated at this time produces a yellow-colored product, such as formazan. Additionally, because the absorbance of the yellow product can be measured at 450 nm, it is possible to quantify the activity of LDH. Prior to measuring the amount of NADH produced by PkLDH, a standard curve was constructed using a known amount of NADH. Different amounts of NADH (0, 2.5, 5.0, 7.5, 10.0, and 12.5 nmole) and substrate mixture were mixed, and the absorbance at 450 nm was measured (Figure 7a). From this result, the equation y = 0.0707x − 0.0053 was obtained, and the R^2^ value was 0.9989. Next, different concentrations of PkLDH (0, 0.01, 0.02, 0.03, 0.06, 0.13, 0.25, and 0.50 nM) and substrate were mixed, and the absorbance was measured at 450 nm after 10 and 30 min reactions (Figure 7b). The LDH activity could then be determined by comparing the amount of NADH generated by the PkLDH reaction with the amount of NADH in the standard curve using the following equation, and the calculated PkLDH activity was 483.9 U/mg.

$$\mathrm{PkLDH}\;\mathrm{Activity}\;(\mathrm{mU}/\mathrm{mg})=\frac{\mathrm{Amount}\;\mathrm{of}\;\mathrm{NADH}\;\mathrm{in}\;\mathrm{sample}\;\mathrm{calculated}\;\mathrm{from}\;\mathrm{NADH}\;\mathrm{standard}\;\mathrm{curve}\;\left(\mathrm{nmole}\right)}{\left(\left(30-10\right)\;\min\right)\times \left(\mathrm{Amount}\;\mathrm{of}\;\mathrm{PkLDH}\;\left(\mathrm{mg}\right)\right)}$$

## 3. Discussion

*P. knowlesi* is the only zoonotic malarial parasite, and the WHO has recently been paying considerable attention to it [1]. Many studies have been conducted on the major malarial parasites, *P. falciparum* and *P. vivax*. In particular, LDH has attracted considerable attention as a major biomarker and therapeutic target for malaria [21,26,27,28]. The amino acid sequence of PkLDH showed a 9.49% difference from that of PfLDH and a 4.75% difference from that of PvLDH. This discrepancy is a sufficient margin to develop specific probes, such as monoclonal antibodies and aptamers, for distinguishing PkLDH. Furthermore, the significant difference in amino acid sequence between PfLDH and PkLDH may be attributed to the fact that *P. falciparum* belongs to the subgenus *Laverania*, unlike other *Plasmodium* spp. that can infect humans. In this study, we optimized PkLDH production using a bacterial expression system. Bacterial expression systems offer many advantages over eukaryotic expression systems in terms of cost, time, yield, and labor [29,30,31].

Recombinant PkLDH was successfully overexpressed and purified from *E. coli* Rosetta(DE3) by inserting the pET-21a(+)-*PkLDH* plasmid. The pET vector series used as an expression vector contains a strong T7 promoter and enables overexpression of target proteins. The pET system has been used to express thousands of different proteins in host cells expressing T7 polymerase [32,33]. The Rosetta(DE3) strain has several advantages over the widely used BL21, BL21(DE3), and BL21(DE3)pLysS strains for eukaryotic protein expression, which were traditionally utilized for recombinant protein expression. Our group has reported successful production of several recombinants using this strain [34,35,36]. The *PkLDH* gene is a eukaryotic gene that contains codons that are rarely used by bacteria. Therefore, we used the Rosetta(DE3) strain specifically designed to enhance the expression of eukaryotic proteins, which does not require additional codon optimization. 

We confirmed that recombinant PkLDH was successfully overexpressed in a saturated state under conditions of 18 °C, 24 °C, and 30 °C. However, we selected induction with 0.1 mM IPTG for 16 h at 18 °C as the optimal overexpression conditions for PkLDH production. Previous studies reported that the solubility of the protein improved when the concentration of the inducer was high at a low culture temperature, as a condition for expressing the protein in a soluble form [37,38,39]. Therefore, protein expression at low temperatures increases protein solubility, which is advantageous for stable protein production. Several reports from previous studies have validated that the protein expression conditions established in this study can stably express PkLDH both structurally and functionally.

We obtained 13.0 mg of PkLDH from a 250 mL culture of *E. coli* Rosetta(DE3). This resulted in a yield of 52.0 mg/L. Additionally, the recombinant PkLDH obtained through this process showed a high enzyme activity of 483.9 U/mg. Many studies have been conducted on the production of PfLDH and PvLDH; to the best of the author’s knowledge, there are only two previous studies on the purification of PkLDH [40,41]. The first study by Singh et al. used the pGEX-6P-1 expression vector and the *E. coli* BL21(DE3) strain to produce PkLDH [40]. In a study by Singh et al., the production yield of PkLDH was 40.0 mg/L and the LDH activity was 350.0 U/mg. Compared to our results, the yield was improved by approximately 30.0%, and the activity was approximately 38.3% higher. Additionally, because the study by Singh et al. overexpressed PkLDH using 0.5 mM IPTG, this study has a cost-effective advantage in that it uses a 5-fold lower amount of IPTG. Lastly, the biggest advantage is that, because the GST fusion protein was not used in this study, the removal of a separate fusion protein (i.e., GST) tag and additional purification of the target protein were not required.

A study by Salim et al. used pET-21a(+) and *E. coli* BL21(DE3) strains and found that the production yield of PkLDH was 0.67 mg/L and LDH activity was 475.6 U/mg [41]. Compared to our results, the LDH activity was similar, but the yield was approximately 77 times higher in our study. We believe that there are several reasons for this. Our results showed that PkLDH of high purity (>99.0%) was obtained in a single step through affinity chromatography, but in the study by Salim et al., size exclusion chromatography was also performed after affinity chromatography. We used LB broth, which is the most widely used broth for bacterial cultures, whereas Salim et al. used Terrific broth. In summary, these results imply that the *E. coli* Rosetta(DE3) strain for expressing eukaryotic proteins greatly aids PkLDH production. If we consider purified PkLDH alone, our research is similar to that of Salim et al. However, it is evident that a substantial amount of PkLDH would be required for the development of drug candidates targeting PkLDH or for the development of bioreceptors, such as monoclonal antibodies and aptamers, specifically binding to PkLDH. Therefore, from this perspective, we anticipate that our methodology could be utilized as a more valuable technique.

## 4. Materials and Methods

### 4.1. Materials

The pUC-IDT-*PkLDH* plasmid was synthesized by Integrated DNA Technologies (Coralville, IA, USA). *PkLDH* was amplified using nTaq-HOT (Enzynomics, Daejeon, Republic of Korea). *Xho*I, *Eco*RI, and T4 DNA ligase were purchased from New England Biolabs (NEB, Ipswich, MA, USA). The DNA extraction kit used was the Exprep^TM^ Plasmid SV (GeneAll, Seoul, Republic of Korea). The gel extraction kit used was the Expin^TM^ Combo GP (GeneAll). The resin used for affinity chromatography was His·Bind^®^ Ni-NTA resin (Merck Millipore, Burlington, MA, USA). For plasmid DNA cloning, DH5α chemically competent *E. coli* (Enzynomics) was used, while protein expression was carried out using Rosetta(DE3) chemically competent *E. coli* (Enzynomics). A BCA protein assay kit for quantitation of protein was purchased from Thermo Fisher Scientific (Waltham, MA, USA). A colorimetric LDH assay kit for measuring the activity of purified PkLDH was purchased from abcam (Cambridge, UK). All chemicals were of molecular biology grade and purchased from Sigma (Saint Louis, MO, USA). 

### 4.2. Gene Cloning of PkLDH and Transformation of E. coli DH5α Strain

The nucleotide sequence of *PkLDH* cDNA was obtained from NCBI (GeneBank No. JF958130.1) and designed with an *Eco*RI restriction site at the 5′-end and an *Xho*I restriction site at the 3′-end for constructing the recombinant plasmid. Subsequently, the *PkLDH* gene was amplified using PCR. The PCR amplification was performed using forward primer (5′-GGC GAA TTC ATG GCA CCA AAA-3′) and reverse primer (5′-GGC CTC GAG AGC TAA TGC CT-3′). PCR was performed in a 20 μL reaction volume containing 1 μL of 10× PCR buffer, 50 μM of each forward and reverse primer, 2 mM of dNTPs, 1 ng of pUC-IDT-*PkLDH*, and 1 U of nTaq-HOT polymerase. The reaction conditions for PCR amplification were as follows: initial denaturation for 1 cycle of 10 min at 95 °C followed by 35 cycles of 1 min at 95 °C, 1 min at 56 °C, and 1 min at 72 °C, and final elongation for 1 cycle of 5 min at 72 °C. The amplified *PkLDH* gene was purified and quantified using a spectrophotometer. The purified PCR product and expression vector pET-21a(+) were digested using the restriction enzymes *Xho*I and *Eco*RI, separated by agarose gel electrophoresis, and extracted from agarose gel. Pure *PkLDH* gene and pET-21a(+) were ligated overnight with 1 U of T4 DNA ligase. The ligated mixture was mixed with competent *E. coli* DH5α and stabilized on ice. Subsequently, heat shock was applied at 42 °C for 1 min, followed by stabilization on ice. After adding 200 μL of super optimal broth with catabolite repression medium to the stabilized DH5α strain, they were cultured at 37 °C with shaking at 200 rpm for 1 h. The transformed DH5α strain culture was spread on a LB agar plate containing 100 μg/mL of ampicillin and incubated at 37 °C for 16 h. A single cell line was obtained from a single colony, and pET-21a(+)-*PkLDH* plasmid was obtained. To confirm successful gene cloning, recombinant plasmid DNA was digested with *Xho*I and *Eco*RI. The *PkLDH* gene sequence of the recombinant plasmid was verified by sequencing with specific primers for the T7 promoter and T7 terminator. The pET-21a(+)-*PkLDH* plasmid was used for protein expression in the subsequent experiments.

### 4.3. Transformation of Rosetta(DE3) Strain and Optimization of PkLDH Expression

Using the same method as the transformation of the DH5α strain, pET-21a(+)-*PkLDH* was inserted into the Rosetta(DE3) strain. The transformed Rosetta(DE3) strain was cultured at 37 °C with shaking at 200 rpm in 250 mL of LB broth containing ampicillin (100 μg/mL) until the optical density (OD) reached 0.7 at 600 nm. The OD_600_ values were measured using an UV-Vis spectrophotometer from OPTIZEN (Daejeon, Republic of Korea). An experiment was performed to determine the optimal conditions for PkLDH expression. First, the Rosetta(DE3) strain was cultured in LB broth containing IPTG at various concentrations, and the saturated concentration at which PkLDH overexpression was confirmed at each culture temperature. IPTG induction was performed under three temperature conditions of 18 °C, 24 °C, and 30 °C, and the culture times were performed for 16 h, 12 h, and 4 h, in that order. The concentrations of IPTG were 0, 0.001, 0.005, 0.01, 0.05, 0.1, 0.5, and 1 mM. The Rosetta(DE3) strain culture that completed the culture under each condition was centrifuged at 13,000 rpm, and the supernatant was removed to obtain the pellet. The obtained pellet was resuspended in PBS and analyzed using SDS-PAGE. Second, the saturation point of PkLDH expression was confirmed for each induction time at the established IPTG concentrations. The Rosetta(DE3) strain was cultured in LB broth containing 0.1 mM IPTG at temperature conditions of 18 °C, 24 °C, and 30 °C, respectively, and bacterial cultures were collected at regular time intervals. The pellets obtained from the bacterial culture were collected by centrifugation at 13,000 rpm and resuspended in PBS. The expression pattern of PkLDH according to the induction time by IPTG was confirmed by SDS-PAGE analysis.

### 4.4. Isolation and Purification of Recombinant PkLDH

To induce overexpression of PkLDH, a transformed Rosetta(DE3) strain was cultured in 250 mL of LB broth containing ampicillin (100 μg/mL) until OD_600_ reached 0.7. The bacterial culture was then incubated at 18 °C for 16 h under 0.1 mM IPTG. The pellet obtained through centrifugation at 7500 rpm for 45 min at 4 °C from the bacterial culture was completely mixed by adding equilibrium buffer (pH 8.0; 500 mM NaCl, 50 mM KH_2_PO_4_, and 5 mM imidazole), 100× Xpert protease inhibitor (GenDEPOT, Barker, TX, USA), and lysozyme (Sigma). This mixture was homogenized using an ultrasonic processor (VCX 130, Sonics & Materials, Newtown, CT, USA) to lyse the cells and release the proteins. The sonication process was performed for 30 min at 30% amplitude for 10 s, followed by a 20 s resting period. The lysate was centrifuged at 9000 rpm for 30 min at 4 °C. The supernatant was pooled and filtered through a 0.22 μm syringe filter from GVS (Bologna, Italy) to prepare for gravity flow affinity chromatography. An empty column was then filled with the Ni-NTA resin. Equilibration was performed by loading 10 times the volume of the resin with the equilibrium buffer. The prepared sample was loaded onto a column to bind the PkLDH protein to the resin. Washing buffer (pH 8.0; 500 mM NaCl, 50 mM KH_2_PO_4_, and 40 mM imidazole) at 30 times the resin volume was passed through the column and washed after loading the lysed bacterial sample. Finally, 10 times the resin volume of elution buffer (pH 8.0; 500 mM NaCl, 50 mM KH_2_PO_4_, and 500 mM imidazole) was passed through the column to elute 6× His-tagged PkLDH. A series of dialyses were performed for stable storage of the eluted PkLDH protein. The first dialysis was performed in PBS (pH 7.4) containing 50 mM imidazole at 4 °C for 16 h. The second dialysis step was performed in PBS (pH 7.4) without imidazole. Finally, a third dialysis step was performed under the same conditions as the second dialysis step to obtain purified PkLDH. After dialysis, the solution containing PkLDH was filtered through a 0.22 μm syringe filter to remove residual impurities.

### 4.5. Biochemical Analysis of Recombinant PkLDH

Purified PkLDH was subjected to a BCA protein assay (Thermo Fisher Scientific), and its concentration was determined by measuring the absorbance at 562 nm. SDS-PAGE was performed to evaluate the purity and molecular weight of the purified PkLDH. Thereafter, 2 μg of PkLDH was prepared by mixing with 4× sample buffer (250 mM Tris-HCl (pH 6.8), 8% SDS, 40% glycerol, 8% β-mercaptoethanol, and 0.02% bromophenol blue), loaded, and separated onto a 12% polyacrylamide gel. To confirm the formation of the PkLDH tetramer, 2 μg of PkLDH was prepared by mixing with 4× native sample buffer (250 mM Tris-HCl (pH 6.8), 40% glycerol, and 0.02% bromophenol blue). The prepared sample was separated on a 10% polyacrylamide gel in the absence of SDS. The purity and molecular weight of PkLDH were determined by comparing the band size with that of a protein marker by Coomassie Brilliant Blue R-250 staining of the gel after electrophoresis.

### 4.6. Analysis of PkLDH Activity

A colorimetric assay was used to measure the activity of purified PkLDH. The experimental procedure was performed according to the manufacturer’s protocol. Briefly, a standard curve for measuring PkLDH activity was constructed using different quantity of NADH (0, 2.5, 5.0, 7.5, 10.0, and 12.5 nmole). The total reaction volume was 100 μL, and 48 μL of assay buffer and 2 μL of substrate mixture were added to 50 μL of each NADH standard. To measure PkLDH activity, the same method as that used in the standard curve experiment was performed. At this time, instead of NADH standards, 50 μL of different concentrations of PkLDH was mixed with 48 μL of assay buffer and 2 μL of substrate mixture. The final concentrations of PkLDH used were 0, 0.01, 0.02, 0.03, 0.06, 0.13, 0.25, and 0.50 nM. The reaction was carried out at 37 °C, and the absorbance after 10 min and 30 min of reaction was measured at 450 nm using a microplate reader from BioTek (Winoosky, VT, USA). The reactions were performed in triplicate.

## 5. Conclusions

In this study, a pET-21a(+)-*PkLDH* plasmid was constructed by inserting the *PkLDH* gene into the expression vector pET-21a(+), and a bacterial expression system was constructed by introducing it into the *E. coli* Rosetta(DE3) strain, which was optimized for eukaryotic protein production. The conditions for efficient PkLDH overexpression in the transformed Rosetta(DE3) strain were optimized. The conditions for overexpression of PkLDH were determined by culturing for 16 h at 18 °C under treatment with 0.1 mM IPTG. The recombinant PkLDH produced in this study was in soluble form and was produced with a high yield of 52.0 mg/L and high purity (>99.0%) by culturing in simple LB broth. In addition, recombinant PkLDH was well formed in the tetramer state and showed an LDH activity of 483.9 U/mg. These results are expected to provide valuable data for combating zoonotic malaria through the development of PkLDH-specific diagnostics and the screening of drug candidates targeting PkLDH.

## Data Availability

Data are contained within the article.

## Abbreviations

BLAST, Basic Local Alignment Search Tool; BCA, bicinchoninic acid; HRP-2, histidine-rich protein-2; IPTG, β-D-1-thiogalactopyranoside; LDH, Lactate dehydrogenase; NCBI, The National Center for Biotechnology Information; Ni-NTA, nickel-nitrilotriacetic acid; PBS, phosphate buffered saline; PCR, polymerase chain reaction; PfLDH, *P. falciparum* lactate dehydrogenase, PkLDH, *P. knowlesi* lactate dehydrogenase; PvLDH, *P. vivax* lactate dehydrogenase; RAT, Rapid antigen testing; SDS-PAGE, sodium dodecyl sulfate-polyacrylamide gel electrophoresis; WHO, World Health Organization.
